# Supplementary material for: Cbp1 and Cren7 form chromatin-like structures that ensure efficient transcription of long CRISPR arrays
Source: Nat Commun. 2024 Feb 22;15:1620. doi: 10.1038/s41467-024-45728-8 (PMC10883916; doi:10.1038/s41467-024-45728-8)
Supplement: Supplementary file 1 — Supplementary Information [file 41467_2024_45728_MOESM1_ESM.pdf]

Supplementary Information

Cbp1 and Cren7 form chromatin-like structures that ensure efficient transcription of long CRISPR arrays

Fabian Blombach, Michal Sýkora, Jo Case, Xu Feng, Diana P Baquero, Thomas Fouqueau, Duy Khanh Phung, Declan Barker, Mart Krupovic, Qunxin She and Finn Werner

Supplementary table 1: Genes within the exta ~28 kb deletion in the original Δcbp1 strain

GeneID	Old locus_tag	New_locus_tag	start	end	strand	annotation	remarks
12417532	SiRe_0633	SIRE_RS03230	607599	608006	-	IS200/IS605 family transposase	
12417533	SiRe_0634	SIRE_RS03235	608153	608584	-	universal stress protein	
12417534	SiRe_0635	SIRE_RS03240	608901	609839	-	peptidase	
12417535	SiRe_0636	SIRE_RS03245	609784	610980	-	MFS transporter	
12417536	SiRe_0637	SIRE_RS03250	611017	611850	-	KaiC domain-containing protein	
12417537	SiRe_0638	SIRE_RS03255	612007	612843	+	undecaprenyl-diphosphatase	
12417538	SiRe_0639	SIRE_RS03260	612864	613799	+	protease	
12417539	SiRe_0640	SIRE_RS03265	614065	614694	+	GMP synthase	
12417540	SiRe_0641	SIRE_RS03270	614922	615443	-	TQO small subunit DoxA domain protein	
12417541	SiRe_0642	SIRE_RS03275	615450	615971	-	TQO small subunit DoxD	
12417542	SiRe_0643	SIRE_RS03280	616768	617958	+	amidohydrolase family protein	
31508441	NA	SIRE_RS13815	617959	618547	-	permease	pseudogene, frameshift
31508442	NA	SIRE_RS13820	618557	619146	-	hypothetical protein	pseudogene, partial
12417545	SiRe_0646	SIRE_RS03300	619182	619715	-	hypothetical protein	
12417547	SiRe_0648	SIRE_RS03305	620185	620883	+	hypothetical protein	
12417548	SiRe_0649	SIRE_RS03310	621000	621803	+	DNA repair ATPase	
12417549	SiRe_0651	SIRE_RS03315	621825	623201	-	amino acid permease	
12417550	SiRe_0650	SIRE_RS03320	623184	623876	+	hypothetical protein	
12417551	SiRe_0652	SIRE_RS03325	623989	625971	+	acetyl-CoA synthetase	
12417552	SiRe_0653	SIRE_RS03330	626028	626558	+	hypothetical protein	
12417553	SiRe_0654	SIRE_RS03335	626611	626925	-	ferredoxin	
12417554	SiRe_0655	SIRE_RS03340	627074	627526	+	hypothetical protein	
12417555	SiRe_0656	SIRE_RS03345	627554	627994	+	hemerythrin	
12417556	SiRe_0657	SIRE_RS03350	628042	629085	+	hypothetical protein	
12417557	SiRe_0658	SIRE_RS03355	629082	629810	+	hypothetical protein	
12417558	SiRe_0659	SIRE_RS03360	630114	630356	+	transcriptional regulator	
12417559	SiRe_0660	SIRE_RS03365	630371	631528	-	peptidase U32	
12417560	SiRe_0661	SIRE_RS03370	631643	632446	-	enoyl-CoA hydratase/isomerase family protein	
31508443	SiRe_0663	SIRE_RS13825	632456	632638	-	ring oxydation complex/ phenylacetic acid degradation	pseudogene, partial
12417562	SiRe_0662	SIRE_RS03375	632622	632945	+	transcriptional regulator	
12417563	SiRe_0664	SIRE_RS03380	632983	633669	+	class II glutamine amidotransferase	
24772322	NA	SIRE_RS03385	633723	633990	+	hypothetical protein	
12417565	SiRe_0665	SIRE_RS03390	633943	635148	-	transposase	

Supplementary table 2: Transcript abundance estimates for chromatin protein encoding genes in *S. islandicus* REY15A and *S. solfataricus* P2.

	S. islandicus REY15A			*S. solfataricus P2*		
Chromatin protein	Locus_tag	TPM replicate 1*	TPM replicate 2*	Locus_tag	TPM replicate 1*	TPM replicate 2*
Alba	SiRe_1125	73486	69176	SSO0962	22508	26079
Alba2	SiRe_1123	2450	2410	SSO6877	2678	3175
Sul7d	SiRe_0668	1525	1090	SSO10610	4727	5594
Cren7	SiRe_1111	1804	1385	SSO6901	3842	4905
Cbp1	SiRe_1547	94	114	SSO0454	289	278

*TPM = transcripts per million

Supplementary table 3: Lethality of *cren7* loss does not depend on *cbp1*. Numbers for colonies obtained, tested and confirmed to be edited are shown for each independent transformation.

Strain	Gene targeted	Colonies obtained	Colonies edited/tested
E233S	SiRe_1111 (*cren7*)	2	0/2
		1	0/1
		0	0/0
	SiRe_0782	34	12/17
Δ*cbp1*	SiRe_1111 (*cren7*)	6	0/6
		30	0/7
		7	0/7
	SiRe_0782	36	4/17

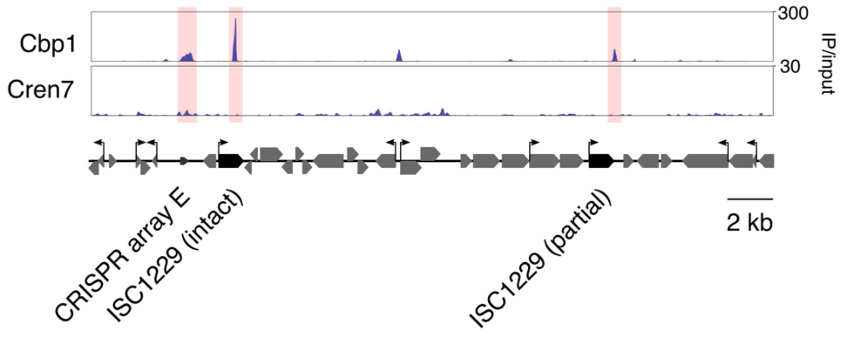

Supplementary figure 1

Occupancy plot of Cbp1 and Cren7 in *S. solfataricus* P2 (ChIP-seq) showing discrete Cbp1 binding sites at ISC1229 transposons versus chromatinization at CRISPR array E. The mean ChIP-seq signal of two biological replicates is shown.

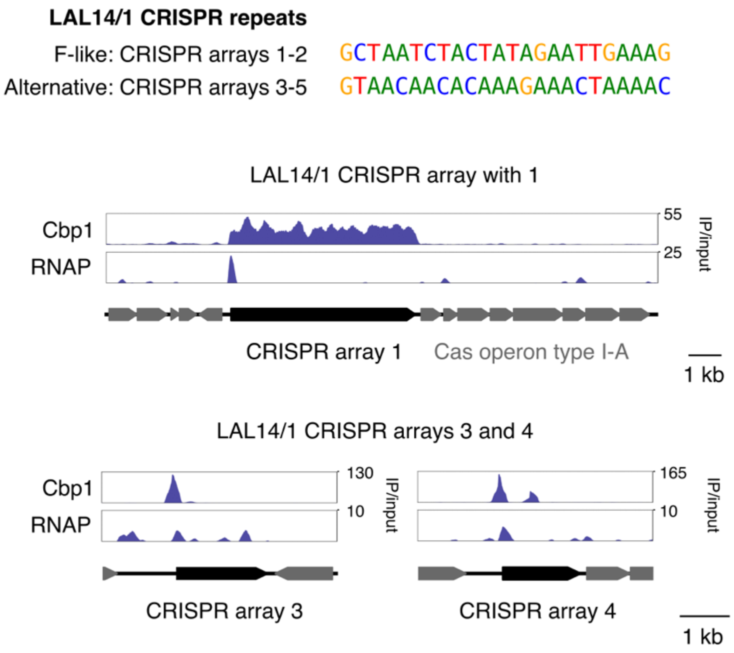

Supplementary figure 2

Cbp1 binds to promoters of CRISPR arrays not chromatinized by Cbp1 in *S. islandicus* LAL14/1. The repeat sequence of F-like CRISPR arrays 1 and 2 compared to the alternative CRISPR repeat sequence of CIRSPR arrays 3 to 5 are shown on top. Below, Cbp1 and RNAP ChIP-seq occupancy (mean of two biological replicates) for CRISPR array 1 with F-like repeat sequences and CRISPR arrays 3 and 4 with an alternative repeat sequence is shown. The F-like CRISPR array 1 is Cbp1-chromatinized. In contrast, CRISPR arrays 3 and 4 do not show Cbp1 binding within the arrays. Instead, they show distinct Cbp1 peaks in their promoter region.

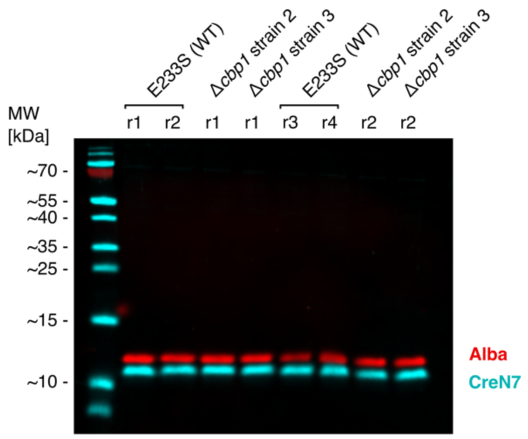

Supplementary figure 3

cbp1 deletion does not affect Cren7 expression levels. Multiplex immunodetection of chromatin proteins Cren7 (cyan) and Alba (red, loading control) in parental strain E233S and two independent *cbp1* deletion strains (Δ*cbp1* strain 2 and strain 3).

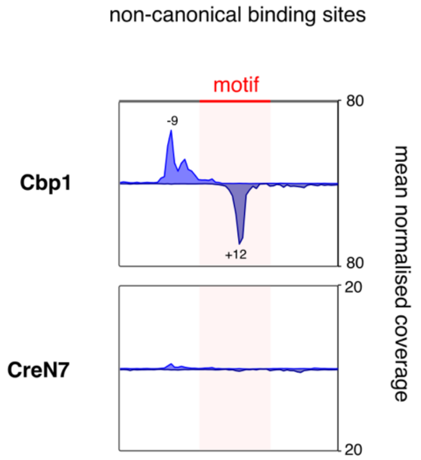

Supplementary figure 4

Cbp1 non-canonical binding sites are similar to CRISPR repeats in terms of ChIP-exo signal. Aggregate plots of Cbp1 and Cren7 ChIP-exo occupancy over 92 non-canonical binding sites in S. solfataricus P2. Signal for the plus and minus strand (relative to CRISPR array orientation) is plotted above and below the x-axis, respectively. The aggregate mean signal was calculated from the geometric mean of two biological replicates scaled to reads per million.

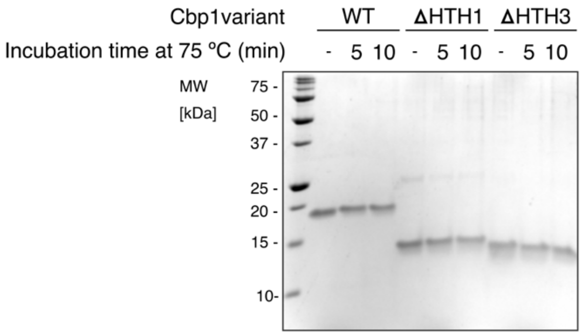

Supplementary figure 5

Heat-stability of Cbp1 HTH-deletion variants. 10 µM Cbp1 or the ΔHTH1 and ΔHTH3 variants were incubated at 75°C for the indicated time and heat-instable, aggregated protein was removed by centrifugation. 10 µl of the heat-stable, soluble protein was detected by SDS-PAGE and Coomassie staining.

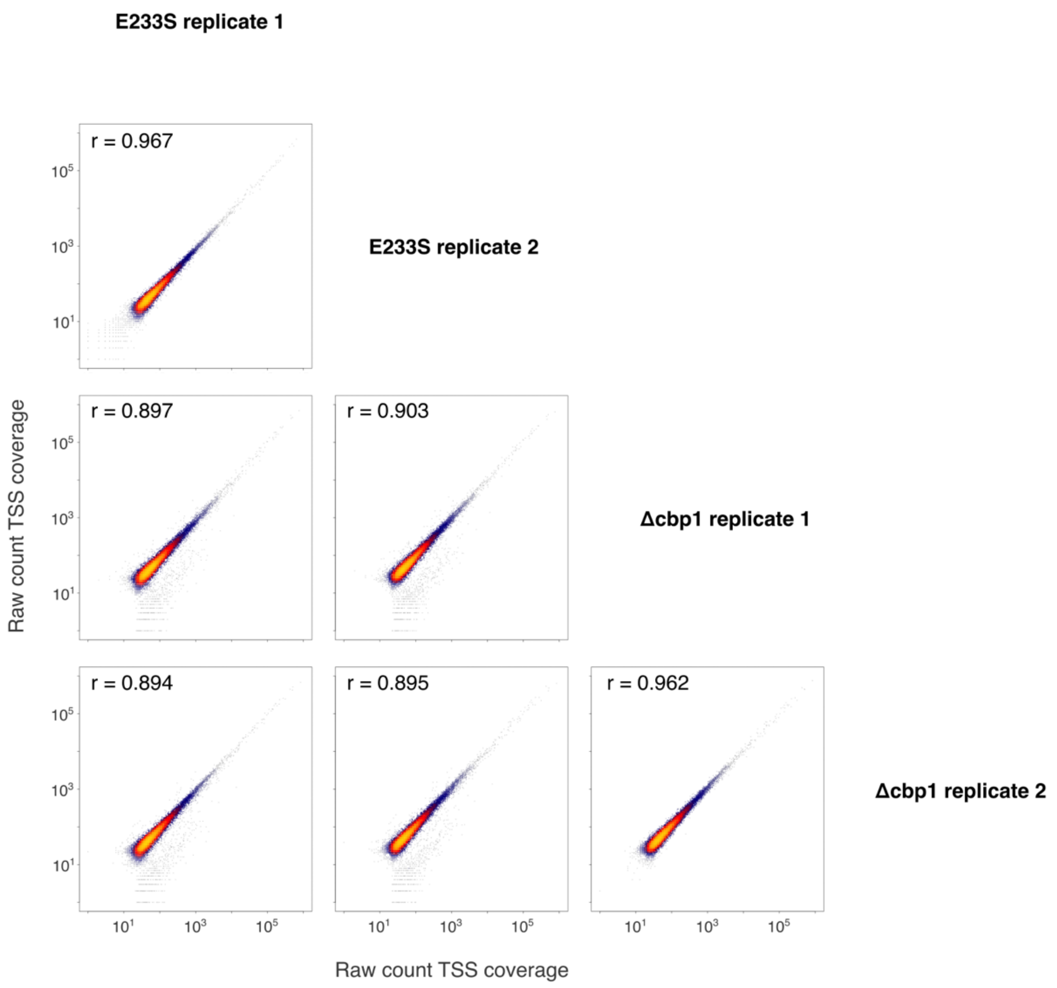

Supplementary figure 6

Correlation of Cappable-seq replicates. The raw count data for 13150 called TSSs are plotted comparing the two E233S replicates and the two *cbp1* deletion strains. The Spearman's correlation coefficient between the samples is indicated on top.

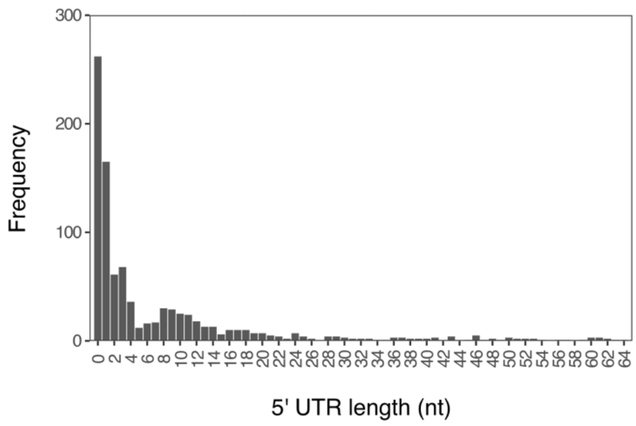

Supplementary figure 7

S. islandicus REY15A transcripts are predominantly leaderless. Histogram showing 5’ UTR length distribution for 934 predicted primary TSSs of operons of coding genes. The maximal 5’ UTR length permitted in the search for primary TSSs was 64 nt equal to the 0.98 quantile of 5’ UTR length distribution in *S. solfataricus* P2.

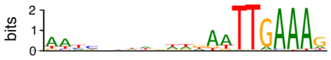

Supplementary figure 8

MEME motif identified for 157 non-canonical binding sites of Cbp1 in *S. islandicus* REY15A.

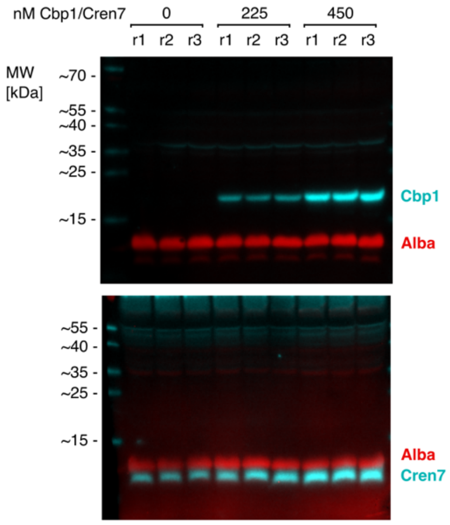

Supplementary figure 9

Multiplex immunodetection of chromatin proteins Cbp1 and Cren7 (both cyan) and Alba (red, loading control) in *S. solfataricus* P2 cell lysate used for transcription assays. 225 nM or 450 nM recombinant Cbp1 and Cren7 were spiked into the cell lysate corresponding to 150 and 300 nM in the cell-free *in vitro* transcription reactions. Three technical replicates are shown.

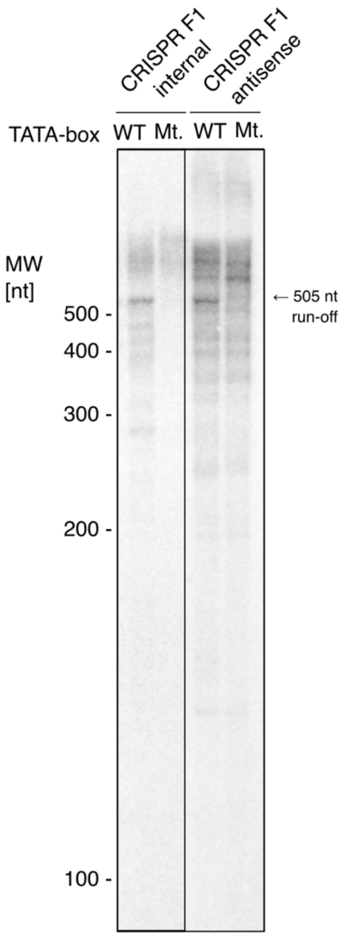

Supplementary figure 10

Control cell-free transcription assay with TATA-box mutations in the internal and antisense promoters from CRISPR array F1.

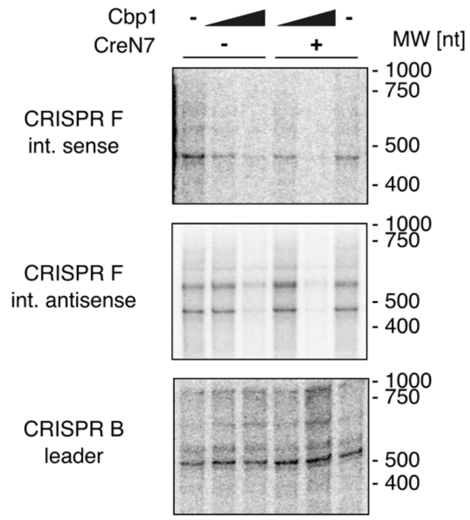

Supplementary figure 11

Cbp1 and Cbp1:Cren7 chromatinization prevents spurious transcription from CRISPR-internal promoters in a competition *in vitro* transcription experiment. The internal sense and antisense promoters in CRISPR F were tested alongside the CRISPR B leader promoter in a single cell-free transcription assay with increasing concentrations of recombinant Cbp1 added (0, 50, 100 nM) and in presence (300 nM) or absence of recombinant Cren7. Reactions containing all three promoters were split for affinity-purification of the radiolabelled transcripts from each of the three promoters and transcripts were resolved on a denaturing polyacrylamide gel. A representative gel of three technical replicates is shown. The expected run-off transcript size was 516 nt for CRISPR B leader and 505 nt for the two CRISPR F internal promoters.

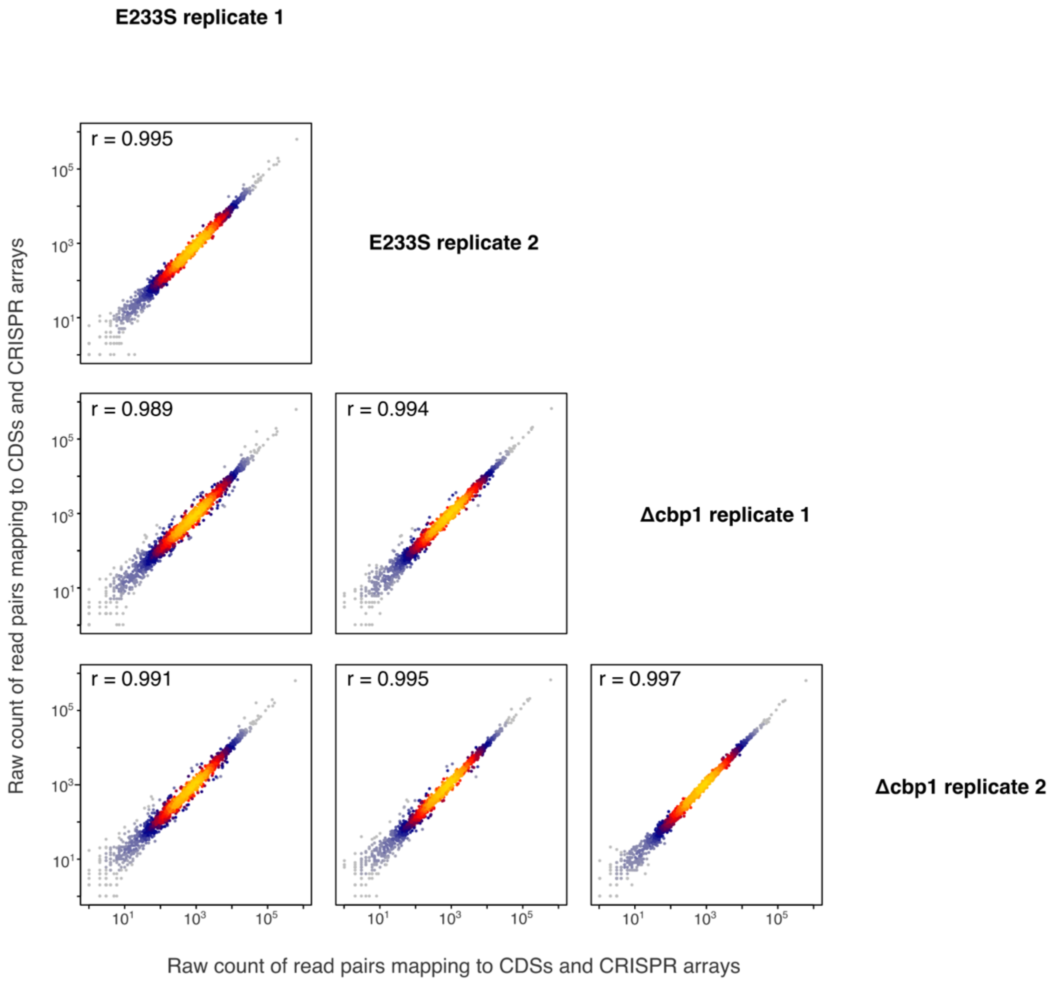

Supplementary Figure 12

Correlation of RNA-seq replicates. The raw count data for read pairs overlapping with 2655 protein encoding coding genes (CDS) with >0 coverage (excluding *cbp1*) and 2 CRISPR arrays are plotted comparing the two E233S replicates and the two *cbp1* deletion strains. The Spearman's correlation coefficient between the samples is indicated on top.

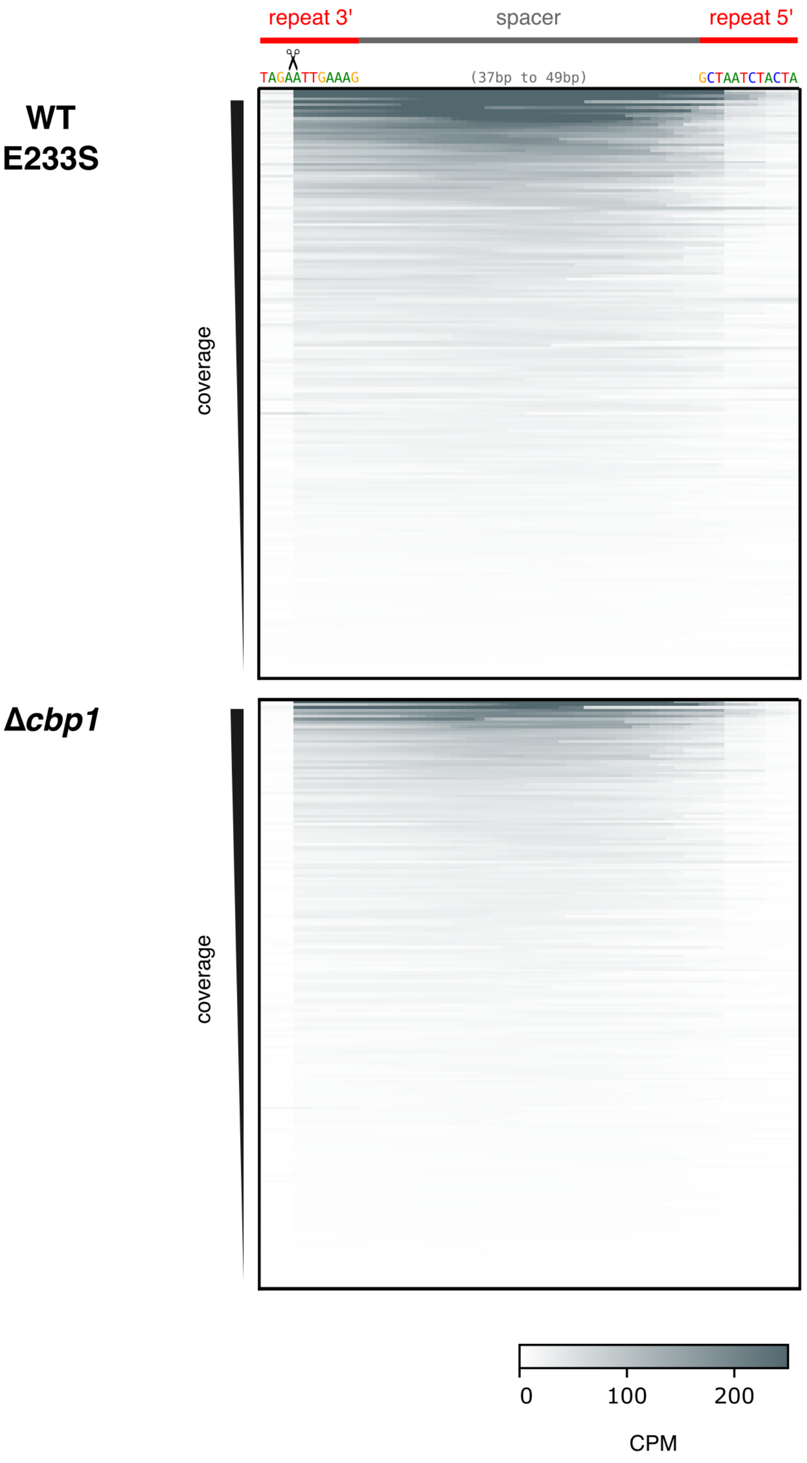

Supplementary figure 13

S. islandicus REY15A RNA-seq coverage over CRISPR arrays is dominated by mature crRNAs. Heatmaps of RNA-seq coverage over CRISPR spacers with 12 bp of the flanking repeat sequences show that the 5′-ends of RNAs are consistent with mature crRNAs after processing by the endonuclease Cas6 and subsequent 3′ processing.

The Cas6 cleavage site is indicated by a scissor symbol. Data represent the arithmetic mean coverage of two biological replicates for WT strain E233Sscaled to counts per million fragments (cpm)

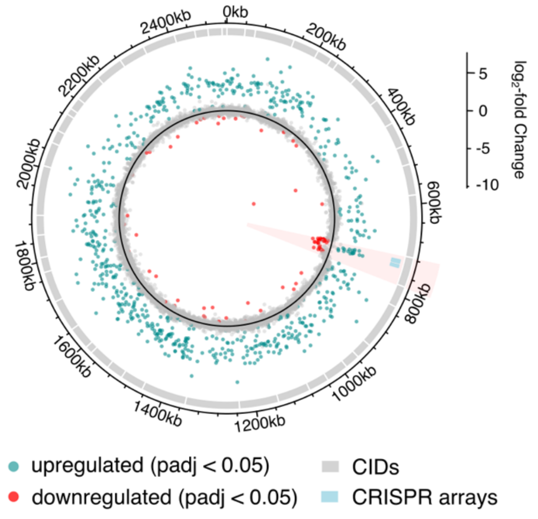

Supplementary figure 14

cpb1 deletion widely affects gene expression in the CID encompassing the two CRISPR arrays in *S. islandicus* REY15A. Circos plot showing log_2-fold changes in relative Cappable-seq signal for 13150 TSSs. Significantly up- and downregulated TSSs (padj < 0.01) are highlighted in green and red, respectively. The ranges of 47 CIDs previously identified by Takemara and Bell [58] are indicated with the CID encompassing the CRISPR arrays highlighted.

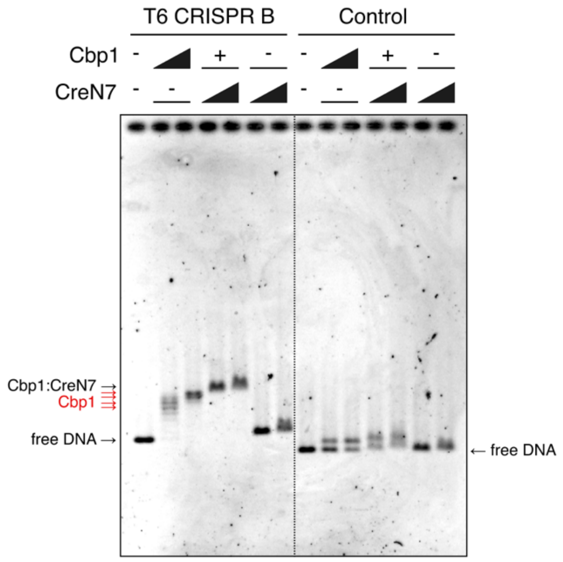

Supplementary figure 15

EMSA experiment testing Cbp1 and Cren7 binding to the T6 CRIPSR B template under in vitro transcription conditions (in absence of RNA polymerase, transcription factors and nucleotides). 150 or 300 nM Cbp1 and 300 or 600 nM Cren7 were tested in the reactions and resolved on a 1.5% agarose gel in 1x TAE buffer and post-stained with ethidium bromide. At lower Cbp1 concentrations, a ladder of bands reflecting recruitment of up to 8 Cbp1 to the CRISPR repeats was visible (highlighted in red) while binding saturates at 300 nM. A ~500 bp dsDNA derived from *Methanocaldococcus jannaschii* with similar AT-content served as control to test binding specificity of Cbp1.

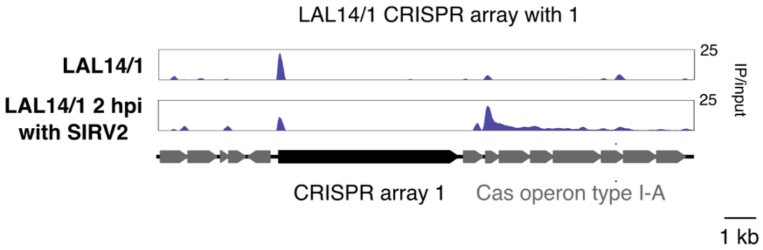

Supplementary figure 16

Occupancy plot of RNAP in *S. islandicus* LAL14/1 (ChIP-seq) showing increased RNAP occupancy on a type I-A Cas operon. The mean ChIP-seq signal of two biological replicates is shown.

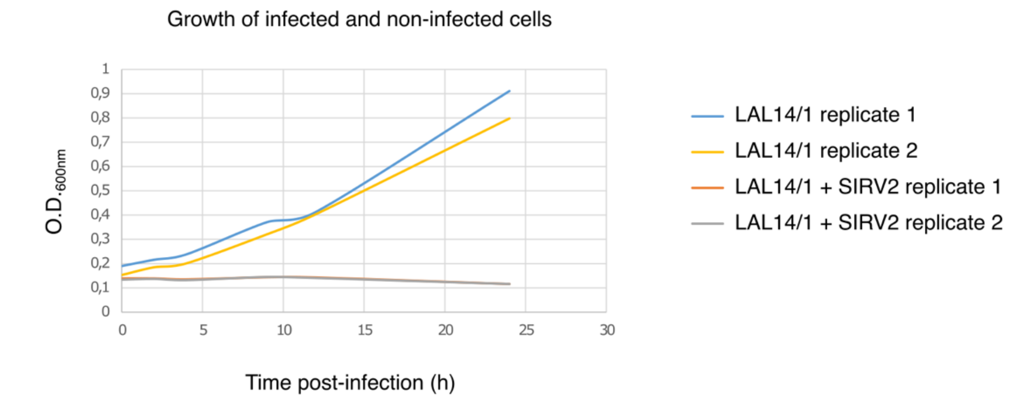

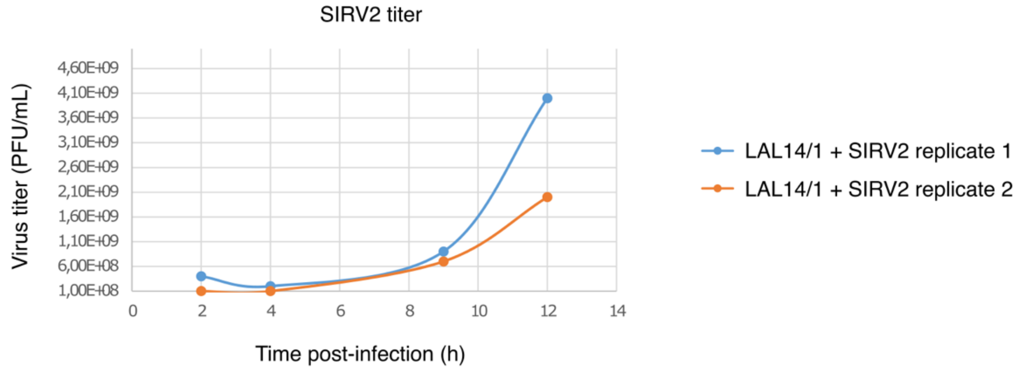

Supplementary figure 17

The upper plot shows the effect of SIRV2 infection on cell growth monitored by measuring optical density at 600 nm for two biological replicates of uninfected and infected cultures, respectively. The lower plot shows the SIRV2 titer over time after infection measured in PFU (plaque forming unit) per mL for the two infected cultures used in ChIP-seq experiments.

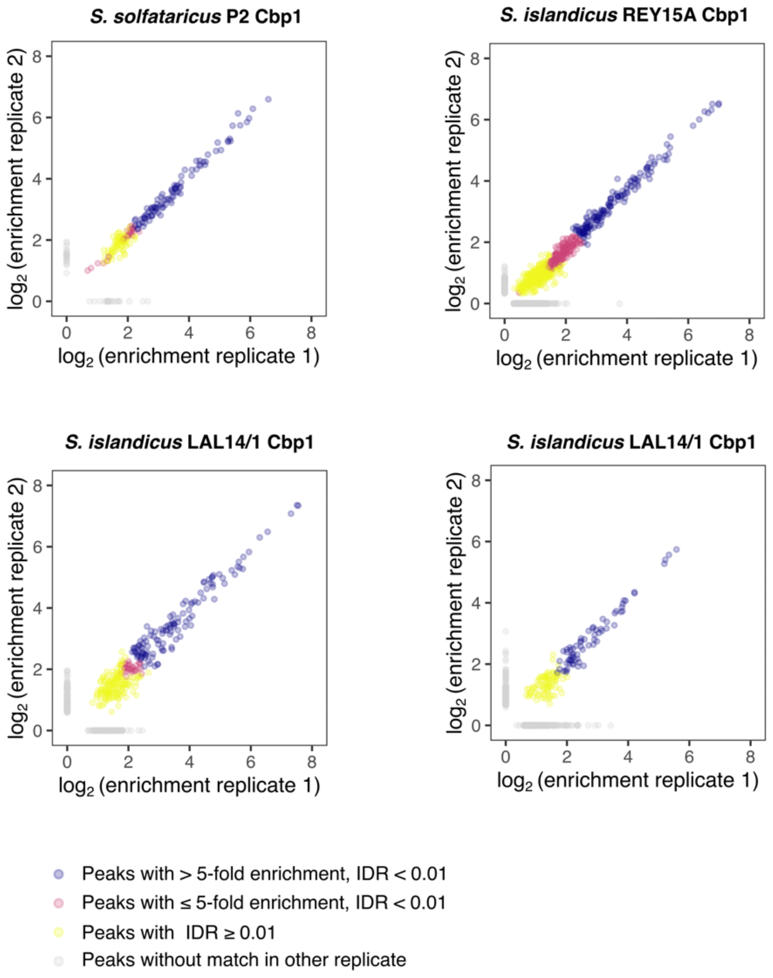

Supplementary figure 18

Scatter plots depicting Cbp1 ChIP-seq peaks called by MACS2 for two biological replicates for each strain (*S. solfataricus* P2, *S. islandicus* REY15A, *S. islandicus* LAL14/1) and growth condition (SIRV2 infection and control). Matched peaks between replicates are coloured based on their IDR score and average enrichment as indicated, unmatched peaks found only in replicate 1 or 2 are depicted in grey along the x- and y-axes, respectively. All subsets of matched peaks showed an R^2 of 0.99 between the log-transformed two replicates.
