# Supplementary material for: Cbp1 and Cren7 form chromatin-like structures that ensure efficient transcription of long CRISPR arrays
Source: Nat Commun. 2024 Feb 22;15:1620. doi: 10.1038/s41467-024-45728-8 (PMC10883916; doi:10.1038/s41467-024-45728-8)
Supplement: Supplementary file 3 — Description of Additional Supplementary Files [file 41467_2024_45728_MOESM3_ESM.pdf]

Description of Additional Supplementary Files:

Supplementary Data 1: Cbp1 ChIP-seq peak calling data for S. solfataricus P2

Supplementary Data 2: Cbp1 ChIP-seq peak calling data for S. islandicus REY15A derivative E233S

Supplementary Data 3: Cbp1 ChIP-seq peak calling data for S. islandicus LAL14/1

Supplementary Data 4: Cbp1 ChIP-seq peak calling data for S. islandicus LAL14/1 2hpi with SIRV2

Supplementary Data 5: List of oligonucleotides and plasmids
