# Supplementary material for: Cbp1 and Cren7 form chromatin-like structures that ensure efficient transcription of long CRISPR arrays
Source: Nat Commun. 2024 Feb 22;15:1620. doi: 10.1038/s41467-024-45728-8 (PMC10883916; doi:10.1038/s41467-024-45728-8)
Supplement: Supplementary file 4 — Supplementary Datasets 1 to 5 [file 41467_2024_45728_MOESM4_ESM.zip › SupplementalFile5.OligonucleotideSequences.pdf]

Supplemental file 5: Oligonucleotide sequences and plasmids

Primers for molecular cloning of *S. solfataricus* P2 *cbp1* and *cren7* and site directed mutagenesis to delete helix-turn-helix motifs

Primer name	Restriction site used for cloning	Sequence**
cbp1 Sso0454 fw	NdeI	GGCG*CAT**ATG***AGCGAGGAAGAAAACATTGA
cbp1 Sso0454 rv	XhoI	GCG*CTCGAG***CTA**AGCAGATGTGGGAGAAGA
cren7 SSO6901 fw	BspHI*	GGC*TC**ATG**A*GTTCGGGTAAAAAACCAGTAAAAGT
cren7 SSO6901 rv	XhoI	GCG*CTCGAG***TTA**TATTGGATAATCATCTGGTAGCT
cbp1 deltaHTH1 SDM fw		AACGATAAAATACAAAAAATCATAGAAATGGGTAA
cbp1 deltaHTH1 SDM rv		GCTCATATGTATATCTCCTTCTTAAAGTTAAA
cbp1 deltaHTH3 SDM fw		ATGGGTTTATATAGACCTATTCGTGA
cbp1 deltaHTH3 SDM rv		TTTTTTAAGAATTCTTAATACGGTATTAAAGTTG

* Compatible cohesive end with NcoI site present in pRSF1-b

** Restriction sites are shown in italic, start and stop codons are shown in bold.

Oligonucleotides used for *cbp1* deletion in *S. islandicus* REY15A

Primer name	Sequence*
KOcbp-SpF	aagAATGATAAAATTCAAAAAATCATAGAAATGGGAAAGCAAG
KOcbp-SpR	agcCTTGCTTTCCCATTTCTATGATTTTTTGAATTTTATCATT
KOcbp-Lf	TTCT*GCATGC*CCATTGACAAACCTAAAATAATCCCT
KOcbp-Lr	TAAGTTCTTTTGCTCCTTGTTCATACATTTTCTTAAC
KOcbp-Rf	GTATGAACAAGGAGCAAAAGAACTTAACATTTCCACTAAT
KOcbp-Rr	TTGT*CTCGAG*GCACATAGGACACCTAATACCATTCAT
KOcbp-checkF	AATTGGCATGGGAATGTACC
KOcbp-checkR	GGGTTCTTTCTATATACTGG

* SpHI and XhoI restriction sites in KOcbp-Lf and KOcbp-Rr, respectively, are shown in italic

Oligonucleotides for dsDNA templates used in EMSA, BS^3 cross-linking and DNase I foot-printing

Oligonucleotide name	Sequence (forward)*	Experiment	Figure panel
CRISPR A repeat 7 20bp flanks	CATCTTCTCTCATCACCTTCGATTAATCCCAAAAGGAATTGAAAGA ATGTATAAAGGTAACCAGG	EMSA	1e
CRISPR D repeat 7 20bp flanks	AACTAACAAATATACGCCTTGATAATCTCTTATAGAATTGAAAGTC AATTTGTGAAACTTGTCC	EMSA	1e
CRISPR F repeat 7 20bp flanks	AAAATTATTGACAAAAATAGGCTAATCTACTATAGAATTGAAAGCT ACACCGATTCCGATACCC	EMSA	1e
IS1229 binding site 20 bp flanks	AACTTCAAGCATAGTGTCAACTAAGATTGCCACGTGAATTGAAAGG ATTCACGTGGCGATTTTGG	EMSA	1e
CRISPR A repeat 7 5bp flanks	CCTTCGATTAATCCCAAAAGGAATTGAAAGAATGT	BS3 crosslinking	1f
T6 promoter negative control	GATAGAGTAAAGTTTAAATACTTATATAGATAGAGTAT	BS3 crosslinking	1f
CRISPR A repeat 1 20bp flanks	GGAAGTATAAAAACACAACAGATTAATCCCAAAAGGAATTGAAAGG AACTAGCTTATAGTTTAGA	EMSA<br>DNase I foot-printing	2b<br>2e

CRISPR A repeat 1 20bp upstream, 2 bp downstream	GGAAGTATAAAAACACAACAGATTAATCCCAAAAGGAATTGAAAGGA	EMSA	2b
CRISPR A repeat 1 20bp upstream, no downstream flank	GGAAGTATAAAAACACAACAGATTAATCCCAAAAGGAATTGAAAG	EMSA	2b
CRISPR A repeat 1 5bp flanks	CAACAGATTAATCCCAAAAGGAATTGAAAGGAACT	EMSA	2c, 2d
CRISPR A repeat 1 A6C/T7G mutation 5bp flanks**	CAACAGATTACGCCCAAAAGGAATTGAAAGGAACT	EMSA	2c
CRISPR A repeat 1 A13/A14C 5bp flanks**	CAACAGATTAATCCCAACCGGAATTGAAAGGAACT	EMSA	2c
CRISPR A repeat 1 A17C/A18C 5bp flanks**	CAACAGATTAATCCCAAAAGGCCTTGAAAGGAACT	EMSA	2c
CRISPR A repeat 1 T19G/T20G 5bp flanks**	CAACAGATTAATCCCAAAAGGAAGGGAAAGGAACT	EMSA	2c
CRISPR A repeat 1 A22C/A23C 5bp flanks**	CAACAGATTAATCCCAAAAGGAATTGCCAGGAACT	EMSA	2c

* All oligonucleotides were annealed to reverse complimentary oligonucleotides to form dsDNA templates.
** Mutations within the repeat sequence are highlighted in red.

The rpo5 control template for the agarose EMSA in Supplementary figure 4 was amplified from a plasmid p1471 bearing a 500 bp region of the *Methanocaldococcus jannaschii rpo5* gene [1] with primers 1522 (GCGGccatggaagaattacaatggctgcag) and 1523 (gcggGGATCCGTTTAACTTGAATTGTTATC).

The CRISPR 4 promoter used in the EMSA shown in Figure 5e was PCR-amplified from plasmid p1687 with primers 2393 and 2394 (see below) before radiolabelling with T4 Polynucleotide Kinase.

Construction of plasmids from which PCR-amplified in vitro transcription templates were generated

In vitro transcription template	Organism	Plasmid backbone	Primer identifier	Cloning method	Resulting plasmid
CRISPR F internal sense promoter, CRISPR F internal antisense promoter	*S. solfataricus* P2	pGEM-T (Promega)	2024 2025	TA cloning	p1636
CRISPR F internal sense promoter TATA-box mutation		p1636	2289 2290 2291 2292	NEBuilder Hifi Assembly (NEB)	p1686

CRISPR F internal antisense promoter TATA-box mutation		p1636	2043<br>2044	Site-directed mutagenesis*	p1649
CRISPR B leader promoter	*S. solfataricus* P2		1333<br>1356	TA cloning	p1421
T6 promoter fusion to CRISPR B	*S. solfataricus* P2	pGEM-T (Promega)	1477<br>1356	TA cloning	p1432
T6 promoter fusion to CRISPR B with repeat 1 randomised		p1432	2323<br>2325	Site-directed mutagenesis*	p1691
T6 promoter fusion to inverted CRISPR B fragment preliminary*	*S. solfataricus* P2		1478<br>1479	TA cloning	p1440
T6 promoter fusion to inverted CRISPR B fragment		p1440	2241<br>2242	Site-directed mutagenesis	p1685

PCR amplification of *in vitro* transcription templates

**In vitro transcription template**	**Plasmid**	**Forward primer identifier**	**Reverse primer identifier**
CRISPR F internal sense promoter	p1636	2301	2302
CRISPR F internal antisense promoter	p1636	2298	2299
CRISPR F internal sense promoter TATA-box mutation	p1686	2301	2302
CRISPR F internal antisense promoter TATA-box mutation	p1649	2298	2299
CRISPR B leader promoter	p1421	2318	2305
T6 promoter fusion to CRISPR B	p1432	2303	2305
T6 promoter fusion to CRISPR B with repeat 1 randomised	p1691	2303	2305
T6 promoter fusion to inverted CRISPR B fragment	p1685	2303	2306
T6 promoter fusion to rpo5 control	p1471 [1] **	2303	1523

* Primers 2303 and 2318 target the pGEM-T and pGEM-T Easy plasmids upstream of the cloned promoter region and were used where the cloned region in the plasmid encompassed less than 100 bp upstream of the TSS.

** p1471 encompasses the T6 promoter fused to a 500 bp region derived from the *M. jannaschii rpo5* gene.

List of primers used for plasmid construction and amplification of *in vitro* transcription templates

Primer identifier	Name	Sequence
1333	CRISPR B promoter fw	AGTAAAGGGTAGTCATGAAGATTTATAAGT
1356	CRISPR B +511 rv	CATAACTGGAGTAATTCCATTCTTTCA
1477	T6 fusion to CRISPR B fw	GATTGATAGAGTAAAGTTTAAATACTTATATAGATAGAGTATAGA TAGGAAGTATAAAAACACAACAGATTAATCC
1478	T6 fusion to inverted CRISPR B fw	GATTGATAGAGTAAAGTTTAAATACTTATATAGATAGAGTATAGA TAGCATAACTGGAGTAATTCCATTCTTTCA
1479	inverted CRISPR B rv	AGGAAGTATAAAAACACAACAGATTAATCC
1523	mja rpo5 BamHI rv	gcggGGATCCGTTTAACTTGAATTGTTATC
2024	CRISPR F fw	TACATTTAATCTAGTAGATACACTT
2025	CRISPR F rv	AAAACAACCGCTTTGCTAATC
2043	CRISPR F int. antisense TATAbox SDM fw	gAAGGCTTTCAATTCTATAGTAGATTAGCTGA
2044	CRISPR F int. antisense TATAbox SDM rv	cAAAGTTACCTGAACCATTATTCTGC
2241	inverted CRISPR B SDM fw	gtaTAACTGGAGTAATTCCATTCT
2242	inverted CRISPR B SDM rv	ttcCTATCTATACTCTATCTATATAAGT
2289	CRISPR F int. sense TATAbox mut HIFI fw	GGTAAAAAAGCTACAACTCTATAGAAGTGCGGGCTTTCAATTCTA TAGTAGATTAGCATATGGAAAGTTTTTGCTCAACCAGTTAGGTAA CAAGTATTC
2290	CRISPR F int. sense TATAbox mut HIFI rv	GAATACTTGTTACCTAACTGGTTGAGCAAAAACTTTCCATATGCT AATCTACTATAGAATTGAAAGCCCGCACTTCTATAGAGTTGTAGC TTTTTTACC
2291	p1636 amplification HIFI fw	TTTGCTCAACCAGTTAGG
2292	p1636 amplification HIFI rv	CCGCACTTCTATAGAGTTG
2298	CRISPR F1 int. antisense -100 fw	gggggGAAAATGAGTTGAAAAAACTTTCAATT
2299	CRISPR F1 int. antisense +500 rv	gggggAGCACATATAGCGTTGGGAAG
2301	CRISPR F1 int. sense -100 fw	gggggAAAACAACCGCTTTGCTAATCT
2302	CRISPR F1 int. sense +500 rv	gggggACCACAACACTTGGAGTTAATGC
2303	pGEM-T -54 fw	gggggACGCGTTGGGAGCTCTC
2305	CRISPR B +511 rv2	gggggCATAACTGGAGTAATTCCATTCTTTCA
2306	inverted CRISPR B rv2	gggggAGGAAGTATAAAAACACAACAGATTAATCC
2318	pGEM-T -49 fw	gggggAGTAAAGGGTAGTCATGAAGATTTATAAGT
2323	CRISPR B repeat1 random fw	CTAGCGATGAAAACATTTTTTGCAGGCTCAACC
2325	CRISPR B repeat1 random T6fusion rv	TTGATCTTTTCAATGTTGTGTTTTTATACTTCCTATCTATACT

Affinity capture oligonucleotides for cell-free transcription assays

Oligonucleotide name	Sequence*
capture CRISPR F internal antisense promoter	TCAACATATTGTCCTTGAATATAATCAGCT
capture CRISPR F internal sense promoter	AGCTACAACTCTATAGAAGTGCGGGCTTTC
capture CRISPR A/B leader promoter	AATCTGTTGTGTTTTTATACTTCCT

* All oligonucleotides were 3'-biotinylated with Biotin-14-dATP (Jena Bioscience) using Terminal deoxynucleotidyl transferase (NEB)

References

1. Ofer, S., et al., *DNA-bridging by an archaeal histone variant via a unique tetramerisation interface*. 2023, Research Square.
2. Blombach, F., et al., *Archaeal TFEalpha/beta is a hybrid of TFIIE and the RNA polymerase III subcomplex hRPC62/39.* Elife, 2015. **4**: p. e08378.
