# Supplementary material for: Cbp1 and Cren7 form chromatin-like structures that ensure efficient transcription of long CRISPR arrays
Source: Nat Commun. 2024 Feb 22;15:1620. doi: 10.1038/s41467-024-45728-8 (PMC10883916; doi:10.1038/s41467-024-45728-8)
Supplement: Supplementary file 5 — Reporting Summary [file 41467_2024_45728_MOESM5_ESM.pdf]

nature portfolio

Corresponding author(s): Finn Werner, Fabian Blombach

Last updated by author(s): Jan 23, 2024

Reporting Summary

Nature Portfolio wishes to improve the reproducibility of the work that we publish. This form provides structure for consistency and transparency in reporting. For further information on Nature Portfolio policies, see our Editorial Policies and the Editorial Policy Checklist.

Statistics

For all statistical analyses, confirm that the following items are present in the figure legend, table legend, main text, or Methods section.

n/a	Confirmed	
☐	☒	The exact sample size (*n*) for each experimental group/condition, given as a discrete number and unit of measurement
☒	☐	A statement on whether measurements were taken from distinct samples or whether the same sample was measured repeatedly
☐	☒	The statistical test(s) used AND whether they are one- or two-sided<br>*Only common tests should be described solely by name; describe more complex techniques in the Methods section.*
☐	☒	A description of all covariates tested
☐	☒	A description of any assumptions or corrections, such as tests of normality and adjustment for multiple comparisons
☐	☒	A full description of the statistical parameters including central tendency (e.g. means) or other basic estimates (e.g. regression coefficient) AND variation (e.g. standard deviation) or associated estimates of uncertainty (e.g. confidence intervals)
☐	☒	For null hypothesis testing, the test statistic (e.g. *F*, *t*, *r*) with confidence intervals, effect sizes, degrees of freedom and *P* value noted<br>*Give P values as exact values whenever suitable.*
☒	☐	For Bayesian analysis, information on the choice of priors and Markov chain Monte Carlo settings
☒	☐	For hierarchical and complex designs, identification of the appropriate level for tests and full reporting of outcomes
☒	☐	Estimates of effect sizes (e.g. Cohen's *d*, Pearson's *r*), indicating how they were calculated

Our web collection on statistics for biologists contains articles on many of the points above.

Software and code

Policy information about availability of computer code

Data collection	No software was used for data collection.
Data analysis	Deep sequencing data analysis:<br>bedtools v2.29.2; samtools 1.10; deeptools 3.4.3; meme 4.11.2; bowtie1.2.3; cutadapt 2.9; macs2 2.1.1<br>R 4.2.1 with the following packages: Biostrings 2.66.0; cowplot 1.1.1; CSAW 1.32.0; edgeR 3.40.2; DESeq2 1.38.3; GenomicRanges 1.50.2; ggplot2 3.4.2; ggbio 1.46.0; idr 1.2; rtracklayer 1.58.0; seqvisr package v0.2.7 (https://doi.org/10.5281/zenodo.6583981); statmod 1.5.0; featureCounts v2.0.6<br><br>Custom code for deep sequencing data analysis:<br>https://github.com/fblombach/Cbp1_public<br><br>Image analysis: ImageQuant TL (Cytiva)<br><br>Sequence alignment: MUSCLE

For manuscripts utilizing custom algorithms or software that are central to the research but not yet described in published literature, software must be made available to editors and reviewers. We strongly encourage code deposition in a community repository (e.g. GitHub). See the Nature Portfolio guidelines for submitting code & software for further information.

Data

Policy information about availability of data

All manuscripts must include a data availability statement. This statement should provide the following information, where applicable:

- Accession codes, unique identifiers, or web links for publicly available datasets
- A description of any restrictions on data availability
- For clinical datasets or third party data, please ensure that the statement adheres to our policy

The sequencing data generated in this study (ChIP-seq, ChIP-exo, RNA-seq and Cappable-seq) and the processed data have been deposited at NCBI GEO under accession code GSE226026 [https://www.ncbi.nlm.nih.gov/geo/query/acc.cgi?acc=GSE226026]. RNA-seq and Cappable-seq data and RNAP, TFB, Spt4/5, aCPSF1 ChIP-seq data for S. solfataricus P2 were obtained from NCBI GEO GSE141290 [https://www.ncbi.nlm.nih.gov/geo/query/acc.cgi?acc=GSE141290] (Ref23). The sequence of ISC1229 was obtained from the ISfinder database (Ref67) [https://isfinder.biotoul.fr/scripts/ficheIS.php?name=ISC1229]. Raw data for plots in Figures 2c, 3b, and 5 and uncropped gel images are provided in the Source Data file.

Research involving human participants, their data, or biological material

Policy information about studies with human participants or human data. See also policy information about sex, gender (identity/presentation), and sexual orientation and race, ethnicity and racism.

Reporting on sex and gender	N/A
Reporting on race, ethnicity, or other socially relevant groupings	N/A
Population characteristics	N/A
Recruitment	N/A
Ethics oversight	N/A

Note that full information on the approval of the study protocol must also be provided in the manuscript.

Field-specific reporting

Please select the one below that is the best fit for your research. If you are not sure, read the appropriate sections before making your selection.

- [x] Life sciences
- [ ] Behavioural & social sciences
- [ ] Ecological, evolutionary & environmental sciences

For a reference copy of the document with all sections, see nature.com/documents/nr-reporting-summary-flat.pdf

Life sciences study design

All studies must disclose on these points even when the disclosure is negative.

Sample size	No statistical method was used to determine appropriate sample sizes. We performed all ChIP-seq, ChIP-exo, RNA-seq and Cappable-seq experiments with two biological replicates. This sample size was chosen to allow statistical tests at an affordable cost. Sample sizes are indicated in the figure legends.
Data exclusions	No data were excluded.
Replication	Cappable-seq and RNA-seq data reproducibility was assessed using Spearman's rank correlation (Supplementary figure 3a). For all ChIP-seq peak calling data (from two biological replicates) the irreproducible discovery rate (Li et al., 2011) was determined. All attempts at replication were successful.
Randomization	No randomization was performed.
Blinding	No blinding was performed.

Reporting for specific materials, systems and methods

We require information from authors about some types of materials, experimental systems and methods used in many studies. Here, indicate whether each material, system or method listed is relevant to your study. If you are not sure if a list item applies to your research, read the appropriate section before selecting a response.

Materials & experimental systems

n/a	Involved in the study	
☐	☒	Antibodies
☒	☐	Eukaryotic cell lines
☒	☐	Palaeontology and archaeology
☒	☐	Animals and other organisms
☒	☐	Clinical data
☒	☐	Dual use research of concern
☒	☐	Plants

Methods

n/a	Involved in the study	
☐	☒	ChIP-seq
☒	☐	Flow cytometry
☒	☐	MRI-based neuroimaging

Antibodies

Antibodies used

All primary antibodies except for Cren7 antibodies were custom antibodies made against recombinant purified protein (Blombach et al., 2021, Nat. Comm. and this paper). Cren7 antibodies were obtained from CUSABIO (CSB-PA502491LA01FPB, Lot O0911A). For immuno-detection of proteins, two different secondary antibodies were used: donkey anti-rabbit IgG Dylight680 (A120-208D6, Lot A120-208D6-5, Bethyl Laboratories) and donkey anti-sheep IgG Alexa488 (A-11015, Lot 1716970, Thermo Fisher).

Validation

All primary antibodies were validated using Western blots (Blombach et al., 2021, Nat Comm. and this paper)

Plants

Seed stocks

N/A

Novel plant genotypes

N/A

Authentication

N/A

ChIP-seq

Data deposition

☒ Confirm that both raw and final processed data have been deposited in a public database such as GEO.

☒ Confirm that you have deposited or provided access to graph files (e.g. BED files) for the called peaks.

Data access links
May remain private before publication.

https://www.ncbi.nlm.nih.gov/geo/query/acc.cgi?acc=GSE226026

Files in database submission

Below is the list of 48 samples deposited in GSE226026. Raw fastq files and bigwig coverage tracks are available for each samples:
GSM7050888 S.islandicus.REY15A.E234.ChIP-seq.Cbp1.replicate1
GSM7050889 S.islandicus.REY15A.E234.ChIP-seq.Cbp1.replicate2
GSM7050890 S.islandicus.REY15A.cbp1.ChIP-seq.Cbp1.replicate1
GSM7050891 S.islandicus.REY15A.cbp1.ChIP-seq.Cbp1.replicate2
GSM7050892 S.islandicus.REY15A.E234.ChIP-seq.CreN7.replicate1
GSM7050893 S.islandicus.REY15A.E234.ChIP-seq.CreN7.replicate2
GSM7050894 S.islandicus.REY15A.cbp1.ChIP-seq.CreN7.replicate1
GSM7050895 S.islandicus.REY15A.cbp1.ChIP-seq.CreN7.replicate2
GSM7050896 S.islandicus.REY15A.E234.ChIP-seq.TFB1.replicate1
GSM7050897 S.islandicus.REY15A.E234.ChIP-seq.TFB1.replicate2
GSM7050898 S.islandicus.REY15A.cbp1.ChIP-seq.TFB1.replicate1
GSM7050899 S.islandicus.REY15A.cbp1.ChIP-seq.TFB1.replicate2
GSM7050900 S.islandicus.REY15A.E234.ChIP-seq.Rpo47.replicate1
GSM7050901 S.islandicus.REY15A.E234.ChIP-seq.Rpo47.replicate2
GSM7050902 S.islandicus.REY15A.cbp1.ChIP-seq.Rpo47.replicate1
GSM7050903 S.islandicus.REY15A.cbp1.ChIP-seq.Rpo47.replicate2
GSM7050904 S.islandicus.REY15A.E234.ChIP-seq.input.replicate1
GSM7050905 S.islandicus.REY15A.E234.ChIP-seq.input.replicate2
GSM7050906 S.islandicus.REY15A.cbp1.ChIP-seq.input.replicate1
GSM7050907 S.islandicus.REY15A.cbp1.ChIP-seq.input.replicate2
GSM7061723 S.islandicus.REY15A.E234.Cappable-seq.replicate1
GSM7061724 S.islandicus.REY15A.E234.Cappable-seq.replicate2
GSM7061725 S.islandicus.REY15A.cbp1.Cappable-seq.replicate1
GSM7061726 S.islandicus.REY15A.cbp1.Cappable-seq.replicate2
GSM7061727 S.solfataricus.P2.ChIP-exo.Cbp1.replicate1

GSM7061728 S.solfataricus.P2.ChIP-exo.Cbp1.replicate2
GSM7061729 S.solfataricus.P2.ChIP-exo.CreN7.replicate1
GSM7061730 S.solfataricus.P2.ChIP-exo.CreN7.replicate2
GSM7061731 S.islandicus.LAL141.ChIP-seq.Cbp1.replicate1
GSM7061732 S.islandicus.LAL141.ChIP-seq.Cbp1.replicate2
GSM7061733 S.islandicus.LAL141.SIRV2.2hpi.ChIP-seq.Cbp1.replicate1
GSM7061734 S.islandicus.LAL141.SIRV2.2hpi.ChIP-seq.Cbp1.replicate2
GSM7061735 S.islandicus.LAL141.ChIP-seq.Rpo47.replicate1
GSM7061736 S.islandicus.LAL141.SIRV2.2hpi.ChIP-seq.Rpo47.replicate1
GSM7061737 S.islandicus.LAL141.ChIP-seq.Rpo47.replicate2
GSM7061738 S.islandicus.LAL141.SIRV2.2hpi.ChIP-seq.Rpo47.replicate2
GSM7061739 S.islandicus.LAL141.ChIP-seq.input.replicate1
GSM7061740 S.islandicus.LAL141.SIRV2.2hpi.ChIP-seq.input.replicate1
GSM7061741 S.islandicus.LAL141.ChIP-seq.input.replicate2
GSM7061742 S.islandicus.LAL141.SIRV2.2hpi.ChIP-seq.input.replicate2
GSM7061743 S.solfataricus.P2.ChIP-seq.Cbp1.replicate1
GSM7061744 S.solfataricus.P2.ChIP-seq.Cbp1.replicate2
GSM7061745 S.solfataricus.P2.ChIP-seq.CreN7.replicate1
GSM7061746 S.solfataricus.P2.ChIP-seq.CreN7.replicate2
GSM7925566 S.islandicus.REY15A.E234.RNA-seq.replicate1
GSM7925567 S.islandicus.REY15A.E234.RNA-seq.replicate2
GSM7925568 S.islandicus.REY15A.cbp1.RNA-seq.replicate1
GSM7925569 S.islandicus.REY15A.cbp1.RNA-seq.replicate2

Genome browser session
(e.g. UCSC)

The genomes are not available in the default genome browser set up. We have made all data tracks including peak calling files and the associated nucleotide sequences and annotations available on Zenodo [10.5281/zenodo.10557037]

Methodology

Replicates

We performed all ChIP-seq, ChIP-exo, RNA-seq and Cappable-seq experiments with two biological replicates

Sequencing depth

Three Saccharolobus species/strains used in this study all have a genome size below 3 Mbp.

For S. solfataricus P2 Cbp1 and Cren7 ChIP-seq samples, we sequenced to a depth of > 0.9 M proper uniquely mapped read pairs after sampling to adjust fragment size distribution to 120 bp average and > 5 M read pairs for the chromatin input processed likewise.

S. islandicus REY15A ChIP-seq samples, we sequenced to a depth of > 0.9 M proper uniquely mapped read pairs after sampling to adjust fragment size distribution to 120 bp average and > 2.5 M read pairs for the chromatin input processed likewise.

S. islandicus LAL14/1 ChIP-seq samples, we sequenced to a depth of > 0.45 M proper uniquely mapped read pairs after sampling to adjust fragment size distribution to 150 bp average and > 5 M read pairs for the chromatin input processed likewise.

S. solfataricus P2 Cbp1 and Cren7 ChIP-exo analysis was based on > 21 M proper uniquely mapped read pairs (equalling total 5' coverage) for each library.
S. islandicus REY15A Cappable-seq data analysis was based on > 11.8 M uniquely mapped reads (equalling total 5' coverage) for each library.

S. islandicus REY15A RNA-seq data analysis was based on > 9.9 M proper uniquely mapped read pairs for each library.

Antibodies

All antibodies used for ChIP-seq other than Cren7 are rabbit antibodies custom produced at Davids Biotechnology (Germany) against recombinant purified proteins. Polyclonal rabbit Cren7 antibodies were obtained from CUSABIO (CSB-PA502491LA01FPB, Lot O0911A).

Peak calling parameters

Peaks were called using MACS2 version 2.2.6 in the BEDPE mode with a cut-off of q = 0.01 and the call-summits subfunction activated. Input data were used as control sample. MACS2 estimated duplicate reads to be within the expected level. Peaks with summits located within the CRISPR arrays were removed for subsequent analysis.

Data quality

Reproducibility of peak calling data was assessed via the Irreproducible Discovery Rate (Li et al, 2011). Details on the size of the reproducible component of peaks and their R^2 are stated in the manuscript.

Software

Custom code has been made available on Zenodo [10.5281/zenodo.10557037]. All other software and version are listed above
