# Supplementary figures and images for: Cbp1 and Cren7 form chromatin-like structures that ensure efficient transcription of long CRISPR arrays
Source: Nat Commun. 2024 Feb 22;15:1620. doi: 10.1038/s41467-024-45728-8 (PMC10883916; doi:10.1038/s41467-024-45728-8)

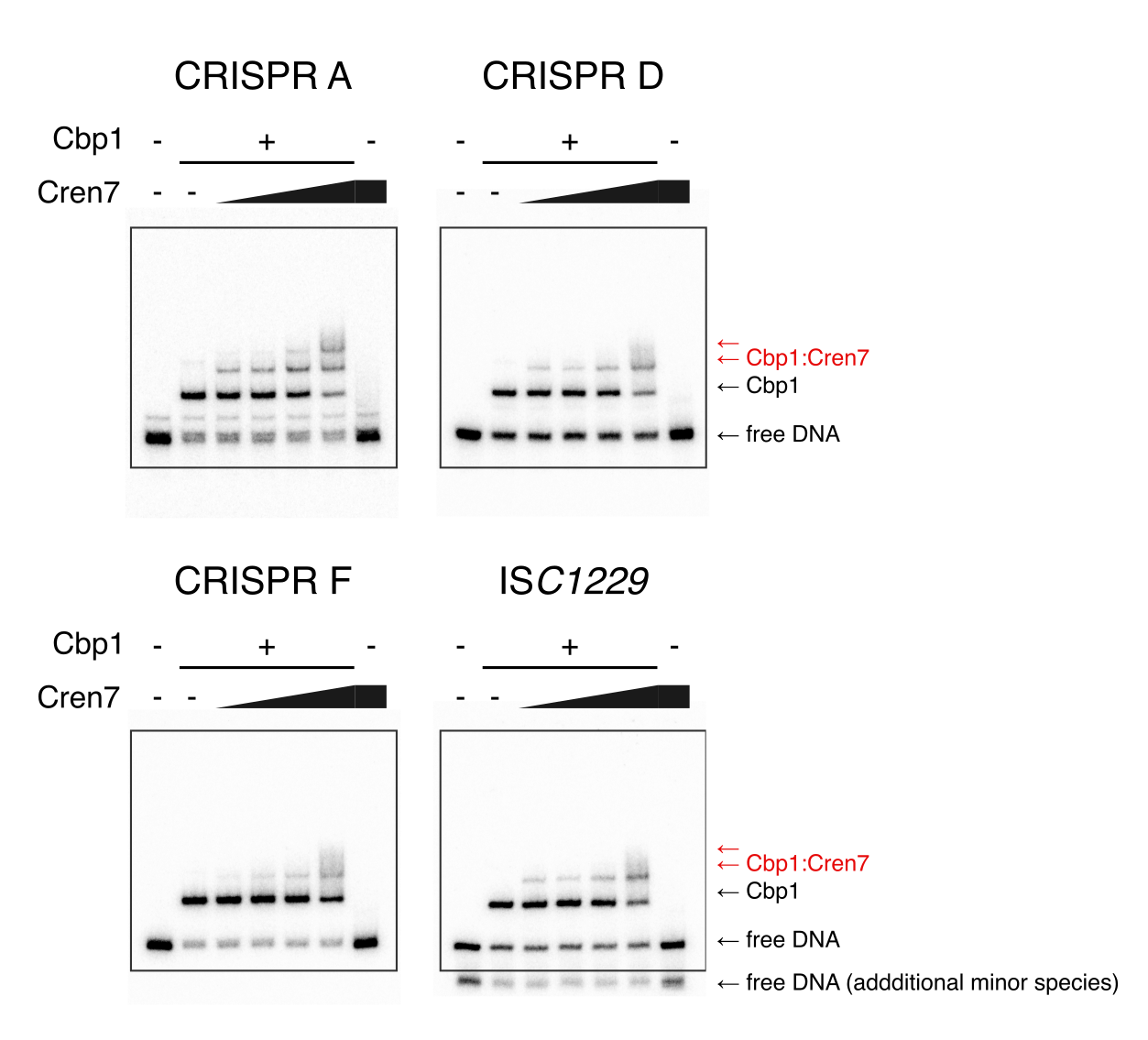

Supplement: Supplementary file 6 — Source Data [file 41467_2024_45728_MOESM6_ESM.zip › Figure1e_uncropped.png]

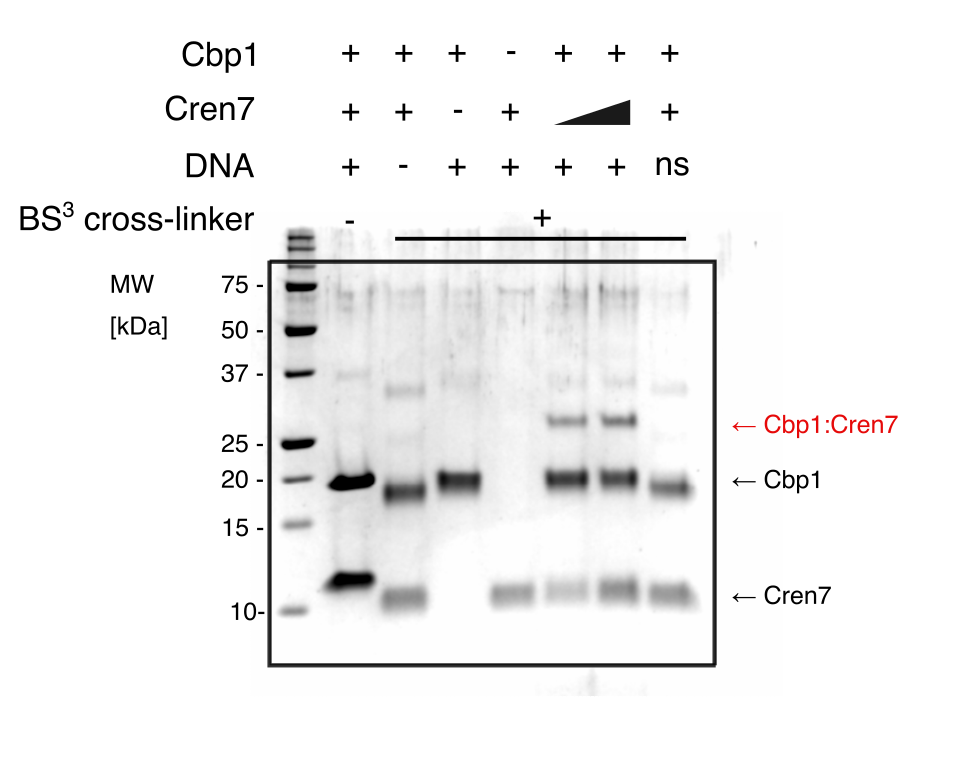

Supplement: Supplementary file 6 — Source Data [file 41467_2024_45728_MOESM6_ESM.zip › Figure1f_uncropped.png]

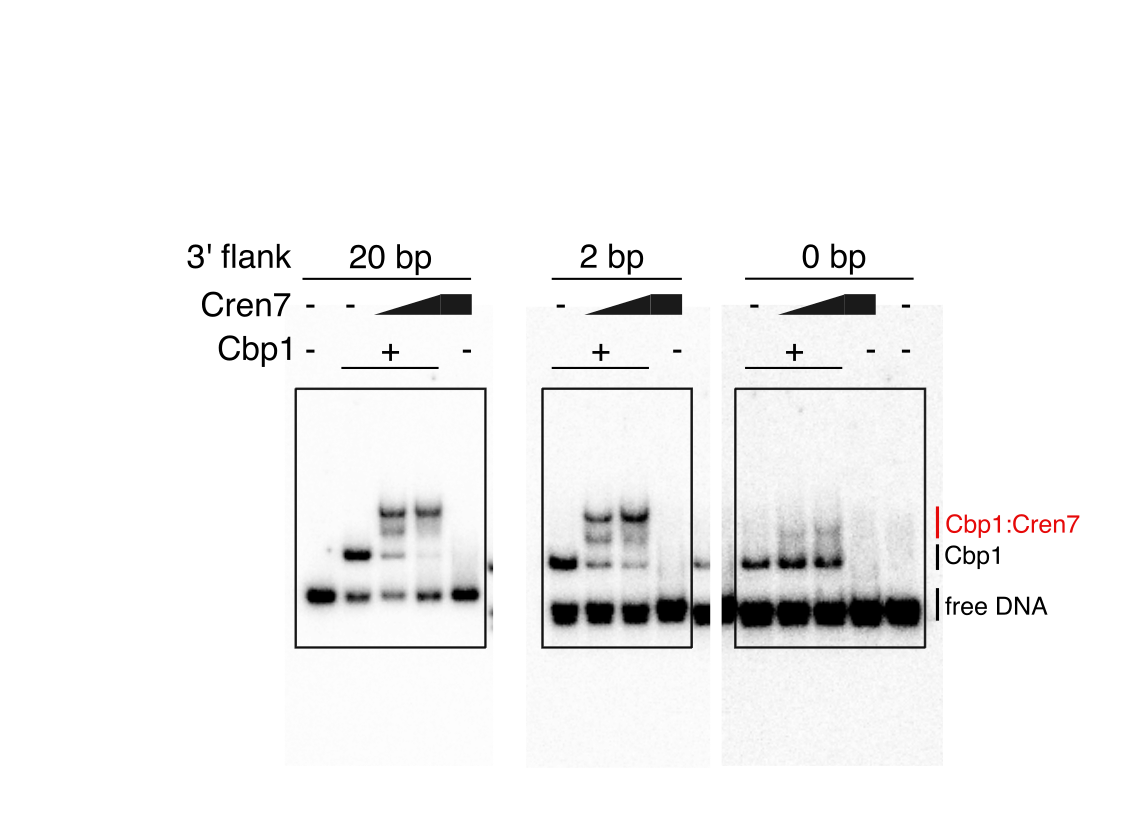

Supplement: Supplementary file 6 — Source Data [file 41467_2024_45728_MOESM6_ESM.zip › Figure2b_uncropped.png]

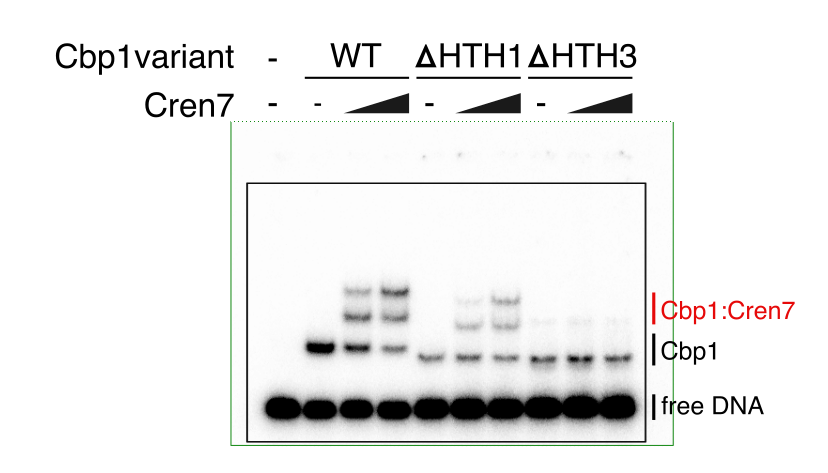

Supplement: Supplementary file 6 — Source Data [file 41467_2024_45728_MOESM6_ESM.zip › Figure2d_uncropped.png]

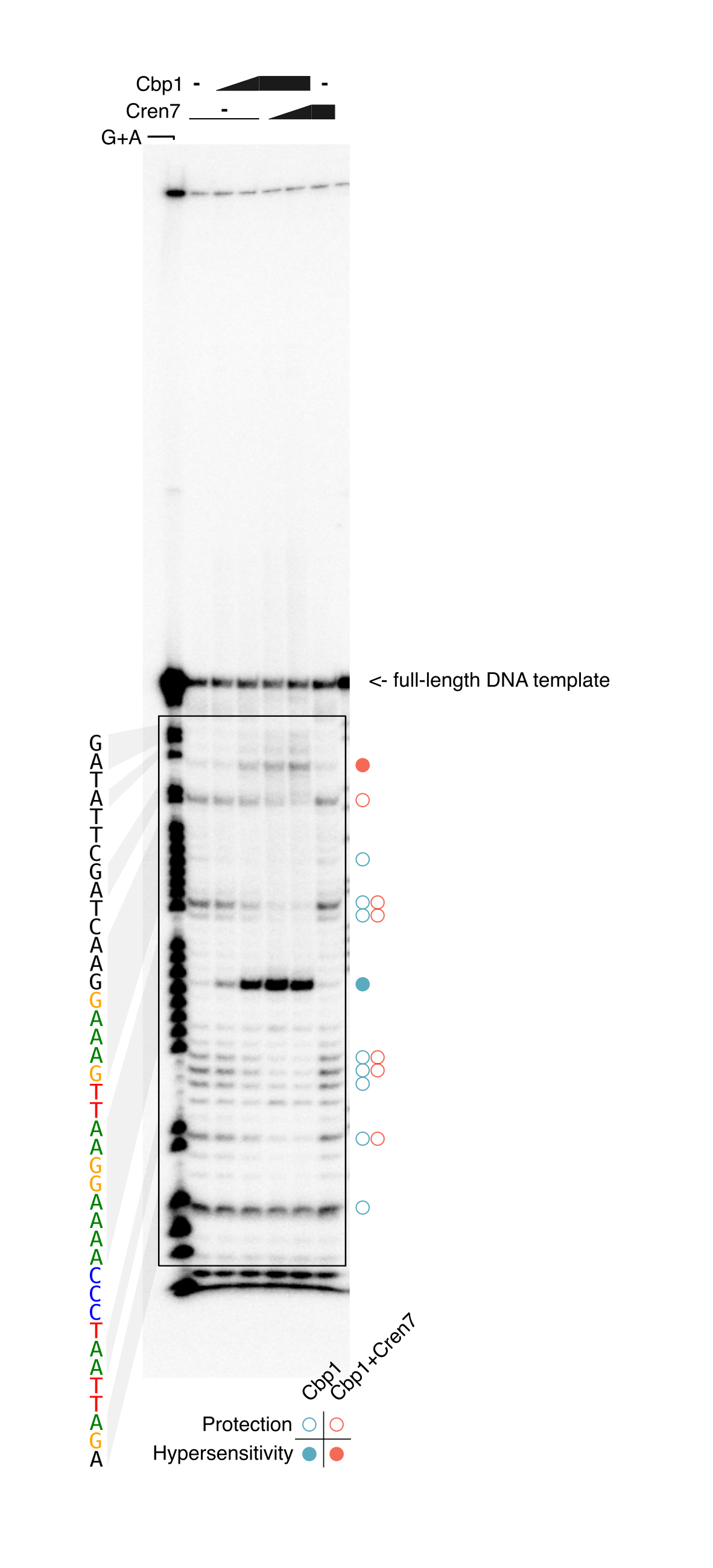

Supplement: Supplementary file 6 — Source Data [file 41467_2024_45728_MOESM6_ESM.zip › Figure2e_uncropped.png]

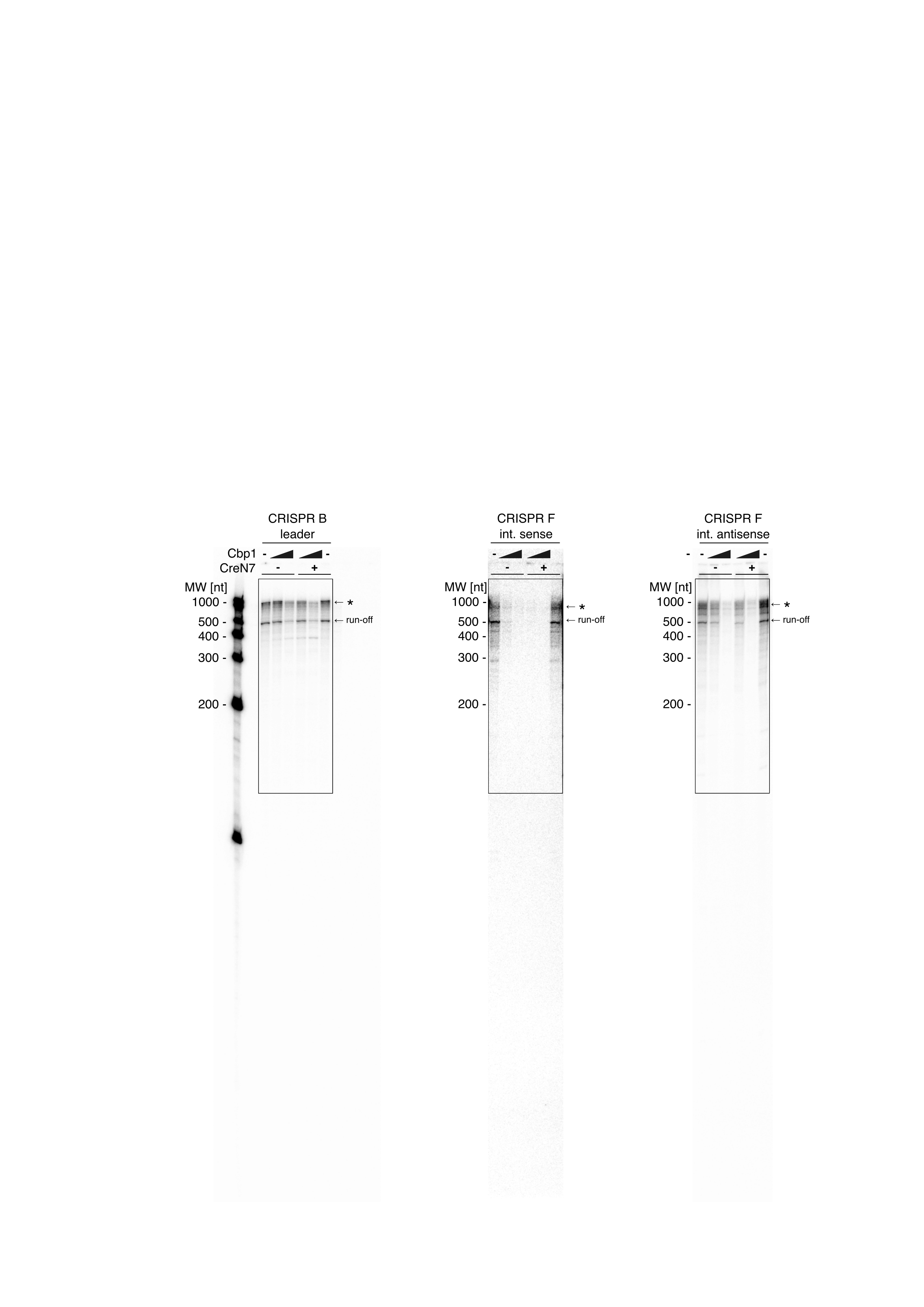

Supplement: Supplementary file 6 — Source Data [file 41467_2024_45728_MOESM6_ESM.zip › Figure3e_uncropped.png]

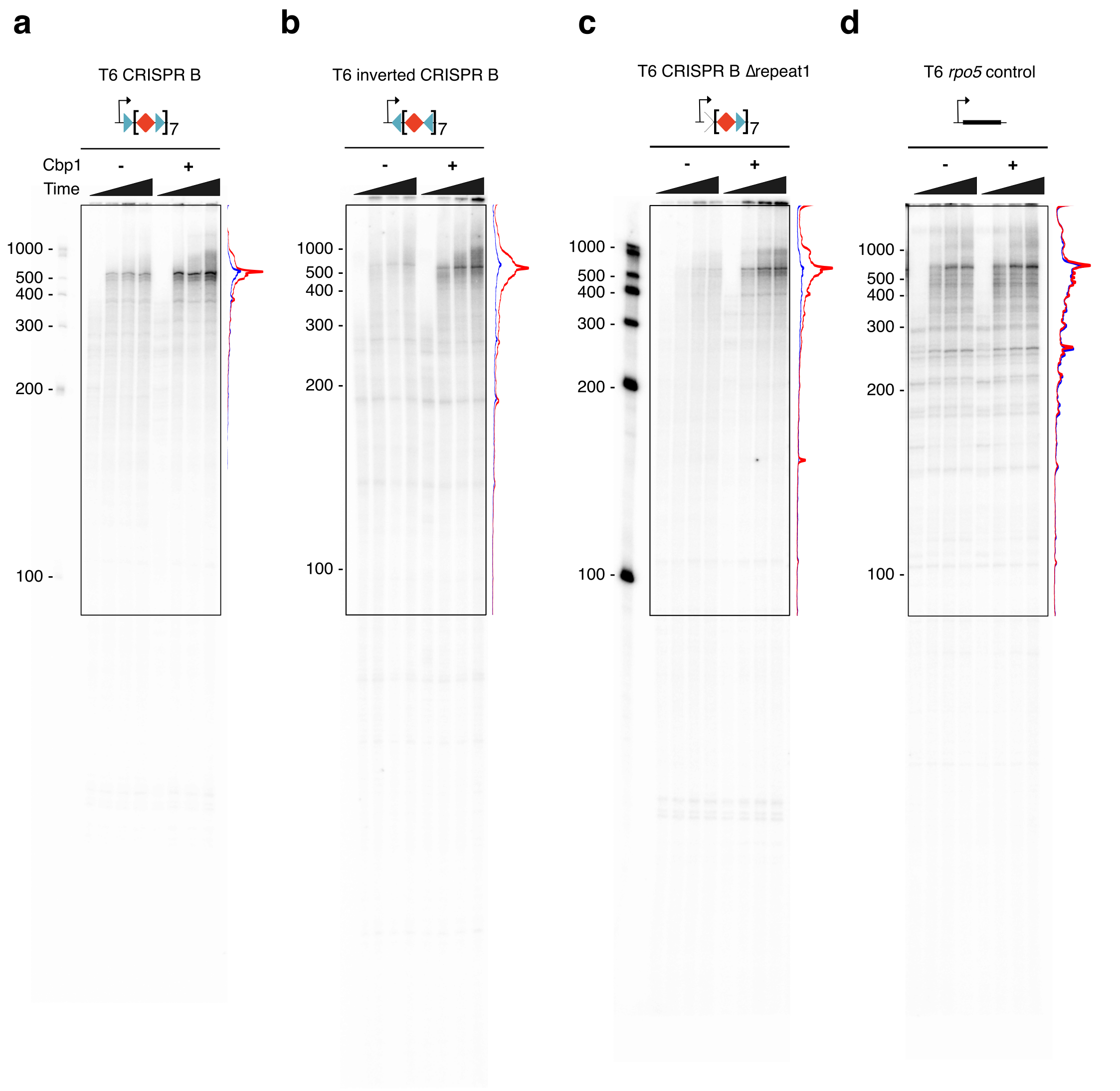

Supplement: Supplementary file 6 — Source Data [file 41467_2024_45728_MOESM6_ESM.zip › Figure4_uncropped.png]

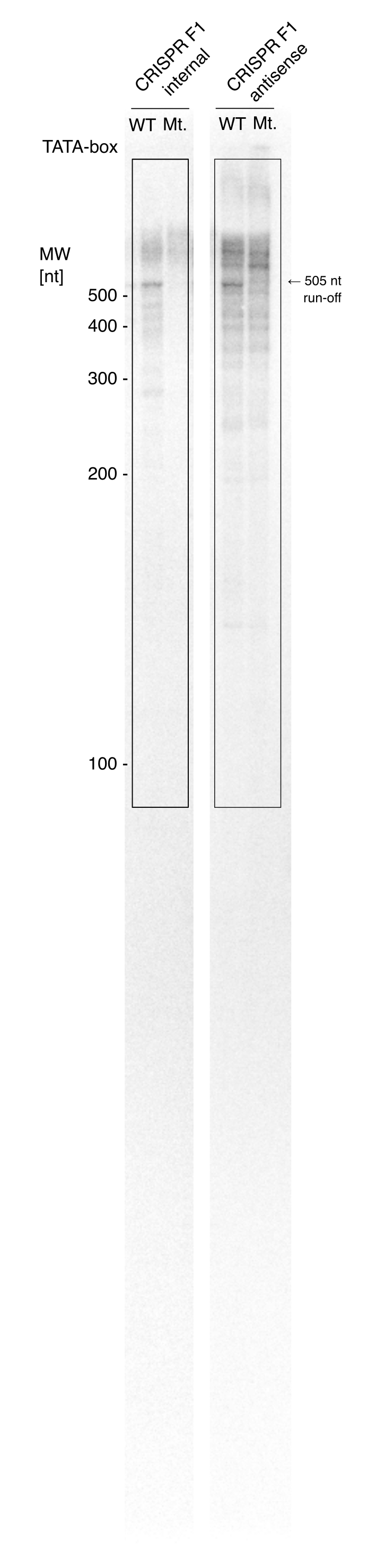

Supplement: Supplementary file 6 — Source Data [file 41467_2024_45728_MOESM6_ESM.zip › SupplementaryFigure10_uncropped.png]

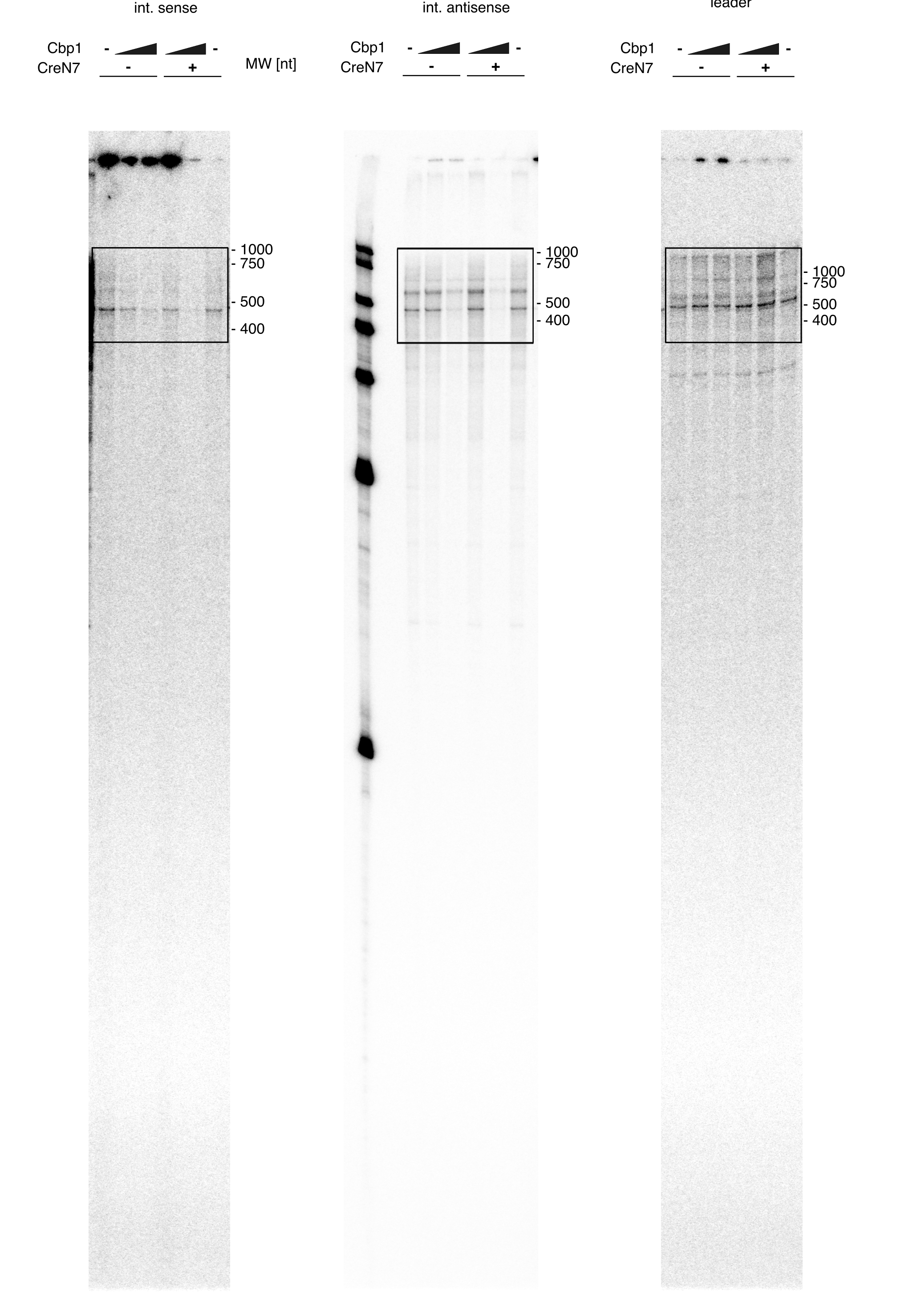

Supplement: Supplementary file 6 — Source Data [file 41467_2024_45728_MOESM6_ESM.zip › SupplementaryFigure11_uncropped.png]
